# New York Letter

**Published:** 1895-08

**Authors:** Ogden C. Ludlow

**Affiliations:** New York


### *CORRESPONDENCE.*

### NEW YORK LETTER.

New York, July 20, 1875.

*To the Editor of the Atlanta Medical and Surgical Journal:*

In my last letter I gave some of the recently-expressed views here regarding the antitoxin treatment of diphtheria. Since then there has been no new public discussion of this subject, although I have been told that the first enthusiasm is rapidly vanishing. We cannot look for any agitation of the matter here until next fall—in reality, the spring of the year, so far as the energy and life of our medical societies is concerned. But this topic is of such vast importance, that at the risk of being a little tedious, I shall venture to refer to a highly important paper just read before the Litchfield County Medical Association by Dr. William H. Welch of Baltimore.

As I listened to his remarks, I could not but feel that it was an exceedingly fair and complete presentation of the subject of the antitoxin treatment of diphtheria. Although a bacteriologist, and not a medical practitioner, he was inclined to give full weight to the views and observations of clinicians. The fact that this treatment, unlike the treatment of tuberculosis with Koch's tuberculin, rested on a sound experimental basis, was particularly urged by him. There was no more impressive laboratory experiment, he said, than the demonstration of the power of the diphtheria antitoxin to prevent and to cure experimental diphtheria. For this reason, it was difficult to understand why it should not exert a similar power against the same poison in the human subject. The preponderance of evidence was apparently in favor of the "vital theory" of the action of antitoxin—that is to say, that it acted through the agency of the cells of the body, and not merely in a chemical way. This being the case, it is easy to understand that, for various reasons beyond our control, as for example, a congenital peculiarity, certain individuals may not be benefited by the serum, and, in any case, we have no right to expect from this, or any other agent acting through cells, absolutely uniform results.

The matter of dosage was, the speaker said, one of vital importance, as was abundantly proved by experiments on animals with the antitoxin, yet there were so many factors beyond our control entering into this question, that the value of the new treatment was necessarily very much restricted in actual practice. Touching upon the statistics of this treatment that had been presented, the reader said that in some instances there was reason for believing that a large number of mild cases of diphtheria, or of pseudo-membranous inflammations of the throat, had been included, and also that erroneous conclusions had been drawn by those who had watched at the bedside for the effects of the serum. As its action was marked by nothing strange or unusual, except a more rapid convalescence, it was impossible to speak positively of the effect of the treatment except after observing a very considerable number of cases. As a rule, there was a lowering of the temperature, an arrest of the local exudation, and a speedy separation of the membrane. He took exception to the statistics of the New York Board of Health, which claimed that from 20 to 40 per cent. of cases believed by the physicians on clinical grounds to be diphtheria, were in fact not diphtheria; he would rather say that these cases formed not over five per cent. of the total number.

Among other interesting statements in the statistical portion of his remarks was this: That he had collected 3,888 cases from various sources in which antitoxin had been used, and the mortality in this series was 18 per cent. Again, out of 2,800 cases treated in France by this method, the mortality was 17 per cent. To make the argument more conclusive, by eliminating the many mild cases of diphtheria, he referred to the results that had been obtained in cases of laryngeal diphtheria requiring tracheotomy or intubation. One observer had collected in the hospital at Marseilles a series of such cases of croup, in which the mortality had been reduced from 89 to 30 per cent. by this treatment. Baginsky reports fifty-three cases of tracheotomy and fifty-four of intubation for diphtheritic laryngeal stenosis, treated with the antitoxin serum, with a mortality of 27.8, and states that during the two months when there was no antitoxin available, the mortality in this same class of cases rose to 62.2 per cent.

In concluding his remarks, Dr. Welch said that he had laid a good deal of stress upon the clinical impressions of good observers, and where these, as they did, spoke with great unanimity of the favorable impression the remedy had produced on them at the bedside, he thought it argued much for the value of the treatment. He considered it to be the duty of every physician to employ the antitoxin wherever practicable; for, while it was by no means a harmless remedy, its effects were not so serious as to warrant the abandonment of the treatment.

Dr. William Henry Porter, of New York, in discussing this subject at the same meeting, said that in his city a large percentage of the cases reported had been called diphtheria because of the presence of Loeffler bacilli, notwithstanding the absence of the usual clinical aspect of the disease. He believed that all those cases which presented the pure clinical phenomena of diphtheria were diphtheria, even though the Loeffler bacilli were not found. He would go even further, and say that these were the cases most likely to prove fatal, as they were commonly cases of mixed infection. As the rational treatment of diphtheria had been vastly improved within the last ten years, it would not do to compare the results of the antitoxine treatment with those secured many years ago. It had seemed to him that the Klebs-Loeffler diphtheria showed a decided natural tendency to recovery.

As the hot summer months bring us often face to face with little children who have been brought suddenly to the verge of the grave by exhausting diarrhea, we are tempted—nay, almost instinctively impelled—to pour into them alcoholic stimulants. But is this wise? Dr. August Seibert, in a paper on "The use of Alcohol in Diseases of Children," read before the Pediatric Section of the Academy of Medicine, replies to this query with a most emphatic negative, and he is not alone in this opinion, although the practice of giving alcoholics freely to little ones, under such circumstances, is a very general one. Dr. Seibert says that alcohol should be avoided in acute and chronic gastro-enteritis, as it only serves to irritate the diseased mucous membrane, and, by setting up an alcoholic gastritis, interferes with the appetite and retards convalescence. Many physicians who favor the use of alcohol do so, he declared, because the increased fullness of the pulse after its administration has deceived them. The so-called "toleration" of children for alcohol was simply due to the fact that the nerve centers had become so diseased by the action of the alcohol, that when this drug was administered freely, it ceased to produce the usual reaction. Where stimulants were required in the diseases of childhood, he preferred, with very few exceptions, to rely on black coffee, tea, cocoa and camphor, with possibly a good dose of some alcoholic stimulant at night. In the discussion on this paper, Drs. W. P. Northrup, Charles G. Kerley, W. L. Stowell and J. E. Winters spoke in much the same strain, but Dr. Northrup stated explicitly that he believed in using it quite freely in cases of severe sepsis, such as were associated with some forms of diphtheria. Dr. Kerley also favored the free administration of alcohol in cases of diphtheria when the heart gave evidence of failing, but a large experience with the pneumonias of children had led him to think that alcohol was rarely called for in pneumonia. As substitutes for alcohol he used strychnine, strophanthus and nitroglycerine. Dr. Winters said that for many years he had but rarely prescribed alcohol for the diseases of children. The author of the paper had done well in pointing out how easy it was to be deceived by the fullness of the pulse into believing that the alcohol was decidedly beneficial. Its deleterious action on the kidneys of young children should also be borne in mind. As the alcohol caused a vaso-motor paresis, aud consequent dilatation of the cutaneous capillaries, the little ones suffered a serious loss of heat and of vital force.

It has often seemed to the writer that physicians are apt to be too fond of the pure science of their calling, and that in their zealous and very laudable pursuit after cold facts with which to build up a complete and well-rounded clinical record, they not infrequently lose sight of the best good of the patient. Those who have had much to do with hospitals can doubtless recall cases in point—some of them ludicrous, and others sad. I remember that at the time that cocaine was introduced here as a local anesthetic, a certain hospital physician, in his anxiety to give the new agent an extensive trial, employed it to relieve the discomfort associated with paracentesis thoracis—in other words, to relieve the pain inflicted by the *single*

thrust of the aspirating needle into the chest; he first inserted the needle of a hypodermic syringe *two or three times*, and injected the solution of cocaine! At another time, a widely-known and respected attending physician of a hospital was so busily engaged in thumping the chest of a poor physical wreck that he did not observe that the patient had passed beyond the reach of human skill until his attention was called to it by one of the house staff. I do not mean to say that in this particular instance the sitting up of the patient in bed and subjecting him to a careful physical examination had much to do with his death, or that it is not very desirable that we should exhaust all possible means for diagnosis before the case comes to autopsy; but I do say that it is much too common, both in hospital and private practice, for persons who are extremely ill and weak to be subjected to prolonged physical examinations. Thus, it is no unusual thing in cases of pneumonia for the physician at almost every visit to make an extended exploration of the chest. Surely he cannot expect such rapid changes in the pneumonic lung, and a proper study of the vital signs and other symptoms should keep him sufficiently informed of the condition of the patient to allow him to safely dispense with many of those exhausting examinations.

Feeling as I do on this subject, it was pleasant to hear recently somewhat similar views expressed by one of our best representatives of that now rapidly-diminishing class—the family physician. I refer to Dr. Andrew H. Smith, who, while progressive, is eminently conservative. In a paper entitled "Some Points in the Physical Examination of the Chest," read by him before the Clinical Society of the New York Post-Graduate Medical School and Hospital, he takes occasion to say that physical examinations are often made unnecessarily, and are then disturbing and injurious to the patient. For instance, he says, it is rarely necessary in cases of pulmonary hemorrhage to make an immediate physical examination of the chest; rest at this time is far more important. Moreover, the examiner may train himself so that in performing percussion he will get as much information from one tap as from the customary three taps—a saving of both time and strength. Referring to the physical exploration of the chest in general, the same physician calls attention to the existence in cases of general muscular weakness of a soft, systolic murmur, having its greatest intensity about half way between the apex and the base of the heart, and not associated with murmurs in the vessels of the neck. It is due to weakness of the papillary muscles. So long as the sound in the second left interspace continues to be distinct, we may be sure that the right heart is doing well. The slightest diminution of the intensity of this pulmonary second sound should be the signal for treatment directed to lightening the labor of the right heart; in other words, a resort to the vascular dilators. Dr. Smith has devised a method of exaggerating the percussion note, which is especially useful in distinguishing slight shades of difference between the apices in cases of suspected lung disease. The thoracic end of the stethoscope is placed in the patient's mouth and his nostrils closed, thus producing an unbroken column of air from the lungs to the drum membrane of the observer's ear. Percussion is then made in the usual way. I can only stop to mention one more of the many valuable practical hints contained in this paper, viz.: that difference in expansion of the two sides of the chest is best observed by seating the patient and, while he is in this position, standing behind him and looking *down* upon the chest. OGDEN C. LUDLOW, M.D.

---

CREOSOTE IN PHTHISIS.—(Dr. C. W. Ingraham, *Medical Age.*)
According to the author, we have in beechwood creosote a remedy productive of much good, and pure beechwood creosote, properly and systematically administered, is without doubt one of the most reliable and standard remedies in the entire pharmacopœia. Failure in its use is due to non-employment of a reliable product, or neglect to prescribe a proper method of administration. A pure beechwood creosote, administered in a proper way, generally gives most successful results.

---

### TALCUM BABY POWDER.

Take 1 gm. of oil of rose-geranium, 25 gm. of boric acid, 50 gm. of corn starch, and 444 gm. of finely powdered talcum, well mixed and sifted. These give a good powder, and should be put in cylindrical sieve-top boxes.—*Ex.*

